# Clinical Study on the Emotional Intervention of Patients with Asymptomatic and Mild Novel Coronavirus (COVID-19)

**DOI:** 10.3390/medicina59050895

**Published:** 2023-05-06

**Authors:** Bo Lu, Wenli Shi, Xunjie Zhou, Deyu Fu, Lei Duan, Xiaoli He, Wenjing You, Junwei Gu, Xinyue Zhang

**Affiliations:** 1 Department of Cardiology, Yueyang Hospital of Integrated Traditional Chinese Medicine and Western Medicine, Shanghai University of Traditional Chinese Medicine, Shanghai 200437, China; 2 Department of Nursing, Yueyang Hospital of Integrated Traditional Chinese Medicine and Western Medicine, Shanghai University of Traditional Chinese Medicine, Shanghai 200437, China; 3 Department of Orthopedics, Yueyang Hospital of Integrated Traditional Chinese Medicine and Western Medicine, Shanghai University of Traditional Chinese Medicine, Shanghai 200437, China; 4 Department of Endocrinology, Yueyang Hospital of Integrated Traditional Chinese Medicine and Western Medicine, Shanghai University of Traditional Chinese Medicine, Shanghai 200437, China; 5 Department of Anesthesiology, Yueyang Hospital of Integrated Traditional Chinese Medicine and Western Medicine, Shanghai University of Traditional Chinese Medicine, Shanghai 200437, China; 6 Department of Traditional Chinese Medicine, Yueyang Hospital of Integrated Traditional Chinese Medicine and Western Medicine, Shanghai University of Traditional Chinese Medicine, Shanghai 200437, China; 7 Department of Surgery, Yueyang Hospital of Integrated Traditional Chinese Medicine and Western Medicine, Shanghai University of Traditional Chinese Medicine, Shanghai 200437, China

**Keywords:** COVID-19, traditional Chinese medicine, Tian Dan Shugan Tiaoxi, emotional intervention

## Abstract

*Background and Objectives:* The aim was to explore the interventional effect of the traditional Chinese medicine (TCM) exercise of Tian Dan Shugan Tiaoxi on the emotions of patients with mild novel coronavirus (COVID-19). *Materials and Methods:* A total of 110 asymptomatic and mildly symptomatic COVID-19 patients from Hongkou Memorial Road Temporary Cabin Hospital and South Renji Hospital were selected between April 2022 and June 2022, and randomly divided into two groups: a control group and an intervention group. There were 55 participants in each group. The control group was treated with Lianhua Qingwen granules, and members of the intervention group were made to practice Tian Dan Shugan Tiaoxi (an exercise that soothes the liver and regulates emotions) every day for 5 days. The Patient Health Questionnaire-9 (PHQ-9), the Generalized Anxiety Disorder questionnaire (GAD-7), and the Symptom Checklist 90 (SCL-90) were used to evaluate the data collected before and after the trial. *Results:* The incidence of anxiety and depression was high in the patients included in this study, at 73.64% and 69.09%, respectively. After intervention, the scores of the Patient Health Questionnaire-9 (PHQ-9) and the Generalized Anxiety Disorder questionnaire (GAD-7) in the two groups had decreased in comparison with those recorded before intervention (*p* < 0.05). The PHQ-9 and GAD-7 scores in the intervention group were significantly better than those of the control group (*p* < 0.05). The factors of somatization, depression, anxiety, hostility, and fear in the SCL-90 in the intervention group were significantly improved after intervention, and generally, better than those in the control group (*p* < 0.05). *Conclusions*: Patients infected with novel coronavirus in shelter hospitals have different degrees of emotional abnormalities. Tian Dan Shugan Tiaoxi can reduce the anxiety and depression of people with mild novel coronavirus, and it can be practiced clinically to improve the recovery rate among infected people.

## 1. Introduction 

Since the outbreak of the novel coronavirus (2019-nCoV) infection characterized by the Omicron strain in Shanghai at the end of February 2022, the cumulative number of people infected with the new strain in Shanghai has exceeded 600,000 [1]. The current epidemic is characterized by rapid spread, strong transmission, and robust stealth. In order to realize the unwavering policy of “dynamic zero elimination” proposed by the state council, Shanghai swiftly built more than 100 shelter hospitals in the form of cabins, with approximately 300,000 total beds, to treat asymptomatic infections and mild cases;, this action has become an effective means to cut off potential channels of infection [2]. The closed environment of the shelter hospitals and the fear of the disease posed a lot of mood changes and caused a lot of emotional problems [3,4,5]. In this regard, how to quickly and effectively alleviate these emotional problems is an urgent hitch to be solved. Traditional Chinese medicine and other traditional methods of effectively dealing with emotions have been widely used in shelter hospitals and contributed immensely to promoting recovery and reducing the rate of severe illness, as well as shortening the duration of illness [6]. Traditional Chinese medicine (TCM) and its emotional and psychological benefits have been used in China for centuries [7,8]. The evaluation of emotions in any disease state is an important point of concern, and COVID-19 is no exception. In a report by Lau et. al. [9], 1063 volunteers comprising 926 hospital workers and 37 laboratory technicians working in high-risk virus laboratories used a TCM herbal extract called Sang Ju Yin plus Yu Ping Feng San. The results showed a zero infection rate in the group that used the TCM in comparison with the control group [9]. The use of TCM in SARS-like infections has shown effective therapeutic outcomes in controlled clinical studies and shortened the disease course [10,11,12]. Studies have shown that COVID-19 results from a disharmony in the bodies qi and fluid, and can cause severe emotional changes as well as introduce toxins [13]. In practice, there have been combinations of TCM with western medicine in COVID-19 aimed at combating the infection and ensuring effective therapeutic outcomes [14,15,16]. Tian Dan Shugan Tiaoxi, an exercise that soothes the liver and regulates emotions, emerged in the Han Dynasty and sought to imitate the movements of bears, tigers, apes, deer, and birds in order to prevent and cure disease and prolong life. This exercise is a technique that combines both movement and stillness and, in summary, the body is positioned naturally upright, the arms are naturally elongated, and the eyes are looking straight ahead. The right knee is bent and set back, with the left leg stretched forward. The left knee is then slightly bent with the left hand stretched forward, and the palm of the left hand faces the right. Both arms are then rotated counterclockwise at the same time while the subject pays attention to the rotation of the waist, hip, and tail sacrum. 

On this basis, this study explored the intervention effect of traditional Chinese medicine—Tian Dan Shugan Tiaoxi—on the emotions of patients with mild novel coronavirus. The data gathered from this research and survey is presented in this article. 

## 2. Materials and Methods

### 2.1. Subject Selection

From April 2022 to June 2022, a total of 110 asymptomatic and mildly symptomatic patients with novel coronavirus infection in the Hongkou Memorial Road shelter and the South Campus of Renji Hospital were selected and undertaken by Yueyang Hospital of Integrated Traditional Chinese and Western Medicine, affiliated with Shanghai University of Traditional Chinese Medicine. The participants were aged between 18 and 70 years old. 

### 2.2. Diagnostic Criteria

Mainly refer to the novel Coronavirus (nCoV) Diagnosis and Treatment Protocol (Trial Ninth Edition) [1]. 

### 2.3. Inclusion and Exclusion Criteria

#### 2.3.1. Inclusion Criteria

The following criteria were utilized for the selection of subjects for inclusion in the research:

(1) Meet the diagnostic criteria for asymptomatic or mildly symptomatic cases in the Diagnosis and Treatment Protocol for novel Coronavirus (nCoV) (Trial Ninth Edition) [1];
(2) Aged between 18–70 years old with no gender limitations;
(3) Voluntarily participate in the study and sign an informed consent form.

#### 2.3.2. Exclusion Criteria

The following criteria were utilized for the rejection of subjects, who thus cannot be included in the research:

(1) Bacterial respiratory tract infection caused by primary or secondary immunodeficiency;
(2) Congenital respiratory malformations;
(3) Congenital heart disease;
(4) Asthma or other chronic airway diseases requiring maintenance treatment, acute respiratory bacterial infections, and severe pulmonary interstitial diseases;
(5) Serious systemic disease (i.e., malignancy, autoimmune disease, or liver or kidney disease) or surgery (e.g., splenectomy or organ transplantation) that the investigator believes may affect the assessment and evaluation of efficacy;
(6) Pregnant or lactating women;
(7) Patients with severe mental illness or cognitive dysfunction/impairment.
(8) Patients with a history of anxiety and depression or taking medications for anxiety and depression.

### 2.4. Intervention Methods

#### 2.4.1. Grouping

The group was randomly divided into an intervention group and a control group, and the treatment course was five (5) days.

#### 2.4.2. Intervention Group

The participants in this group were given Lianhua Qingwen granules(Yiling Pharmaceutical, Shijiazhuang, China) (twice a day, one packet at a time) + Tian Dan Shugan Tiaoxi (an exercise that soothes the liver and regulates emotions). The intervention was performed by the participants under the supervision of a health worker. 

#### 2.4.3. Control Group

The participants in this group were given Lianhua Qingwen granules (twice a day, one packet at a time). These granules are a traditional Chinese medicinal compound whose main function is to eliminate inflammation.

### 2.5. Tian Dan Shugan Tiaoxi (An Exercise That Soothes the Liver and Regulates Emotions)

Based on the adaptation of the “Deer Opera” in Wuqinxi, combined with the “Xu” character exercise of the “Liu Zi Jue” health preservation method, it has the effect of soothing the liver, relieving depression, and regulating emotions.

### 2.6. Observation Indicators

#### 2.6.1. Patient Health Questionnaire-9 (PHQ-9)

One of the depression detection scales recommended by the World Health Organization (WHO) is compiled based on the diagnostic and statistical manual IV (DSM-IV) diagnostic criteria for major depressive episodes, and is widely used for the diagnosis and efficacy evaluation of depression. It consists of 9 entries, with each item scoring 0–3 points for a total of 0–27 points, where 0 is never at all and 3 is almost every day. The scoring scale is as follows: 0–4 points for no depression; 5–9 points for mild depression; 10–14 points for moderate depression; and >15 points for severe depression [17].

#### 2.6.2. Generalized Anxiety Scale-7 (GAD-7)

This scale is mainly used for the self-assessment of anxiety and consists of 7 items. The score setting and scoring standard of the items are the same as that of the PHQ-9 [18].

#### 2.6.3. Symptom Checklist-90 (SCL-90) Scale

This checklist scale is widely used, including 9 symptom factors such as somatization, interpersonal sensitivity, paranoia, terror, depression, obsession, anxiety, hostility, and psychosis, in addition to 90 other items. The score of each factor is between 1 and 5, with a total score range of 90–450. The higher the value is, the more serious the psychological problem is, and the SCL-90 has good reliability and validity in the normal population [19].

### 2.7. Evaluation Method

An online survey was conducted by Questionnaire Star. All patients completed the scale before and after the intervention in order for practitioners to evaluate the intervention effect. The psychometric characteristics, which involve reliability, validity, and norming, were all adhered to and concluded such that they fit the goal of the research.

### 2.8. Statistical Analysis

SPSS 26.0 software (IBM SPSS Statistics for Windows, Version 26.0. Armonk, NY, USA: IBM Corp.) was used for data processing, and *p* ≤ 0.05 was considered to be statistically significant. When the measurement data met the normal distribution, they were described as mean ± standard deviation. When the variance was homogeneous, the independent sample t test was used for comparison between the two groups. Repeated measurement data were compared by repeated measurement variance analysis. The enumeration data were expressed by frequency, component ratio and rate, and the comparison of intergroup rates was performed by Chi-square test.

## 3. Results

### 3.1. General Information

Fifty-five (55) participants, each, were selected for the intervention group and the control group. The general data of the two groups of patients are shown in Table 1 and Table 2, which are statistically comparable (*p* < 0.05). The sociodemographic characteristics considered showed more females (*n* = 64; 58.2%) infected than males (*n* = 46; 41.8), many married couples (*n* = 82; 74.5%) were infected, and a high number of infections were transmitted by close relations at home (*n* = 43; 39.1%). 

### 3.2. Psychological Status of COVID-19 Patients

According to the results of the GAD-7 and the PHQ-9, the incidence of anxiety and depression among the 110 patients who participated in this study was 73.64% and 69.09%, respectively. Generally, the incidence of anxiety and depression among the female participants was higher than that in males, but with no statistically significant difference (*p* > 0.05), as shown in Table 3.

Compared with the domestic norm (reflexive societal perspective), which includes both a subjective process of self-conscious inquiry and the study of social behavior with reference to theories, the scores of each factor of the SCL-90 in the studied patients were higher than the norm in the five factors: somatization, depression, anxiety, hostility, and fear. This is shown in Table 4.

### 3.3. Comparison of Psychological Status between Groups of COVID-19 Patients

At the time of enrollment, the incidence of anxiety (GAD-7) and the incidence of depression (PHQ-9) in the control group were 72.72% and 67.27%, respectively. The incidence of anxiety and the incidence of depression in the intervention group were 74.54% and 63.63%, respectively. There was no significant statistical difference in the incidence of anxiety and depression between the two groups (*p* > 0.05), which were comparable, as shown in Table 5.

In addition, there was no significant difference between the two groups in the scores of each factor of the SCL-90 at the time of enrollment, which were comparable, as shown in Table 6.

### 3.4. Comparison of Psychological Status of Patients with COVID-19 before and after Intervention

After intervention, anxiety (GAD-7) and depression (PHQ-9) in the two groups were significantly improved compared with before intervention (*p* < 0.05). After intervention, there were also significant differences in the GAD-7 and PHQ-9 scores between the two groups (*p* < 0.05). It was observed that the improvement of anxiety (GAD-7) and depression (PHQ-9) in the intervention group was enhanced in comparison with the control group, as shown in Table 7.

The comparison of SCL-90 factors between the two groups before and after intervention showed that anxiety factor in the control group were improved after intervention compared with before intervention (*p* < 0.05), and somatization, depression, anxiety, hostility, terror/fear, and other factors in the intervention group were significantly improved after intervention in comparison with before intervention (*p* < 0.05). In a nutshell, members of the group that received intervention were more improved than those of the control group (*p* < 0.05), as shown in Table 8.

### 3.5. Comparison of the Hospitalization Days for COVID-19 Patients

The number of days in hospital for the control group and that for the intervention group were 9.67 ± 2.18 and 9.01 ± 2.63 days, respectively. The number of days in hospital in the intervention group was lower than that in the control group, but there was no statistically significant difference (*p* > 0.05). See Table 9.

## 4. Discussion

Lianhua Qingwen granules are a traditional Chinese medicinal compound whose main function is to eliminate inflammation. This TCM has been used in COVID-19 treatment from the time when the outbreak began [20,21]. The mechanism of action of the granules is presented by Shen et. al., [22] which was beneficial in serving as a comparative work for our research. Since the outbreak in Shanghai at the end of February this year (2022), the novel coronavirus represented by the Omicron strain has shown stronger infectivity and faster transmission, with the cumulative number of COVID-19 infections in Shanghai now exceeding 600,000. Shelter hospitals have played a pivotal role in controlling the spread of the epidemic. However, due to the special environment of the shelter hospitals and the fear of the disease itself, patients in these hospitals show different degrees of emotional disorders. There are reports that [5] in 2020, patients in shelter hospitals in Wuhan had different mental health problems, with the incidence of anxiety and depression among these patients at about 72.73% and 71.72%, respectively [3]. In a recent survey of patients in shelter hospitals in Shanghai, 261 cases (97.4%) were detected with psychological symptoms, among which the detection rates of depression, neurasthenia, fear, obsessive-anxiety, and hypochondria were 80.6%, 69.8%, 89.6%, 75.4%, and 41%, respectively [23]. In contrast, the depression rate of the general population in the non-pandemic period was 5.9% [24], and anxiety 5.3% [25], thus showing a very heightened rate of both depression and anxiety after infection. The rate of symptom detection was similar to those of anxiety (73.64%) and depression (69.09%) in the patients included in this study. As a source of stress, the short-term outbreak of the epidemic can undoubtedly lead to psychological trauma [26]. In the event of an infection and in an isolated state during treatment, with patients being unable to contact relatives and get timely social support, in addition to worrying about how and when the infection will be cured, can cause psychological impairment to a certain extent, mainly manifested as excessive anxiety, fear, sadness, depression, and somatic symptoms [27,28]. In this regard, timely intervention of the emotional problems of patients in shelter hospitals is particularly critical and of great concern. The role of traditional Chinese medicine in emotional intervention has been established and was also found to improve the abnormal mood and sleep quality of drug addicts [29]. A typical example is Taijiquan, which has been proven to have certain effects on improving the emotions of different people [30].

There is a Chinese quote which states that “Lingshu (Native god)” made all things connected to the heart and the mind and all emotional activities are firstly generated by the heart. Nonetheless, there is a connection between the heart, mind, and liver, and anything that affects the heart thus directly affects the mind and the liver, and vice versa [31]. One school of thought believes that “the heart hides the spirit” and “the liver hides the soul”, and among the five internal organs, the heart and liver are most closely related to emotions. In *the Secret Scriptures of Suwen Linglan*, it is said, “The heart is the official of the monarchs and commands the gods to come out and the liver is the official of the generals and brings forth a plan”. Between the two, the mother (heart) and child (liver) are born together, and the liver qi (energy) is unobstructed, making the heart qi (energy) peaceful. If the liver qi (energy) is stagnant, then the heart qi (energy) will be insufficient and unable to refresh the mind. As such, emotional activities are governed by the heart and regulated by the liver. Only when the function of the liver is normal is the liver qi (energy) calm and able to function with no obstruction. Only when qi and blood are in harmony can the spirit and emotions of a person be peaceful and harmless; otherwise, there will be an emotional abnormality called “Liver Qi Deficiency”, which leads to fear and anger [32,33].

Therefore, the regulation of emotions often starts with the “liver”. The main intervention method used in this study is the Tian Dan Shugan Tiaoxi (an exercise that soothes the liver and regulates emotions). This exercise was first created by Professor Fu Deyu and has the effect of assisting in lowering blood pressure [34]. It is widely used in people with hypertension and coronary heart disease (CHD) [34,35], and has achieved satisfactory curative effects thus far. In addition, it combines the “Xu” character of the “Liu Zi Jue” health-preserving exercises. “Liu Zi Jue” is a widely spread set of health-preserving exercises. Among them, the “Xu” character exercise exerts the effect of soothing the liver and regulating qi (energy) through exhalation and breathing guidance.

In this study, the scores of the GAD-7 and the PHQ-9 in the intervention group were decreased compared with those recorded before enrollment, and were more improved than those in the control group. Similar observations have been made in research that used TCM [36]. The results obtained suggest that the intervention of calming and soothing the liver and toning the breath through exercises can improve the anxiety and depression of COVID-19 patients in shelter hospitals. In research by Sartarao et. al., [37] an assessment on the emotional health and wellbeing of individuals was done using the scores of the GAD-7 and the PHQ-9, and many of the wellbeing indicators used in this work were also used in ours. Correspondingly, somatization, depression, anxiety, hostility, terror/fear, and the other factors of the SCL-90 in the intervention group were more improved compared with those in the control group. The results suggest that Tian Dan Shugan Tiaoxi has the effect of regulating emotions. Conversely, although patients in the intervention group showed a trend of shortening the course of the disease, this trend lacked statistical significance, which may be related to the fact that patients in both groups had taken Lianhua Qingwen Granules (which have been proven to shorten the time frame of infection and result in patients testing negative within a shorter period of time) [38]. 

## 5. Conclusions

In summary, patients infected with the novel coronavirus in shelter hospitals have different degrees of emotional abnormalities. Tian Dan Shugan Tiaoxi (an exercise that soothes the liver and regulates emotions) can improve the anxiety and depression of these people, and its use is worthy of clinical promotion.

## Figures and Tables

**Table 1:** Basic information of 110 COVID-19 patients.

Characteristics	Number of Cases	Constituent Ratio (%)
Gender		
Male	46	41.8
Female	64	58.2
Age		
18 to 29	12	10.9
30–39	26	23.6
40–49	17	15.5
50 to 59	27	24.5
60–69	28	25.5
Education Level		
Junior High School and below	31	28.2
High school/technical secondary school	34	30.9
College	22	20.0
University degree or above	23	20.9
Marital Status		
Single	28	25.5
Married	82	74.5
Place of Infection		
Home	43	39.1
Workplace	22	20.0
Medical institution/facility	4	3.6
A public place	29	26.4
Elsewhere	12	10.9

**Table 2:** General data of the two groups of patients ($\bar{x}\pm s$).

	No of cases (example)	Age	Gender		Height (cm)	Body Weight (kg)	Number of Cases of Underlying Diseases (Number)
			Male	Female			
Control Group	55	48.07 ± 14.05	20	35	167.05 ± 7.43	60.89 ± 12.31	0.56 ± 0.60
Intervention Group	55	47.03 ± 16.10	26	29	167.65 ± 8.43	62.42 ± 12.35	0.58 ± 0.74

**Table 3:** Pre-intervention gender distribution of anxiety and depression in COVID-19 patients (*n* (%)).

Survey	Male	Female	*p* Value
GAD-7 ≥5 points	33 (71.74)	48 (75.00)	> 0.05
<5 points	13 (28.26)	16 (25.00)	
PHQ-9 ≥5 points	30 (65.22)	46 (71.88)	> 0.05
<5 points	16 (34.78)	18 (28.12)	

**Table 4:** Pre-intervention scores of SCL-90 factors in patients with COVID-19 ($\bar{x}\pm s$).

	Average Score	Domestic Norm	*t* Value	*p* Value
Somatization	1.79 ± 0.53	1.37 ± 0.48	4.09	<0.05
Obsessive Compulsive symptoms	1.67 ± 0.56	1.62 ± 0.58	1.62	>0.05
Interpersonal Relationships	1.66 ± 0.53	1.65 ± 0.51	0.95	>0.05
Depression	1.85 ± 0.49	1.50 ± 0.59	3.39	<0.05
Anxiety	2.47 ± 0.55	1.39 ± 0.43	6.18	<0.05
Hostility	1.63 ± 0.46	1.48 ± 0.56	2.84	<0.05
Terror/Fear	1.44 ± 0.40	1.23 ± 0.41	2.47	<0.05
Paranoia	1.39 ± 0.49	1.43 ± 0.57	0.76	>0.05
Psychosis	1.34 ± 0.47	1.29 ± 0.42	0.71	>0.05

**Table 5:** Comparison of anxiety and depression between groups of COVID-19 patients (*n* (%)).

Survey	Control Group	Intervention Group	*p* Value
GAD-7 ≥5 points	40 (72.72)	41 (74.54)	>0.05
<5 points	15 (27.28)	14 (25.46)	
PHQ-9 ≥5 points	37 (67.27)	35 (63.63)	>0.05
<5 points	18 (32.73)	16 (36.37)	

**Table 6:** Comparison between groups of SCL-90 factors in patients with COVID-19 ($\bar{x}\pm s$).

	Control Group	Intervention Group	*t* Value	*p* Value
Somatization	1.85 ± 0.50	1.72 ± 0.56	1.26	>0.05
Obsessive Compulsive symptoms	1.66 ± 0.56	1.69 ± 0.53	0.89	>0.05
Interpersonal Relationships	1.68 ± 0.59	1.65 ± 0.51	0.98	>0.05
Depression	1.86 ± 0.43	1.84 ± 0.56	0.30	>0.05
Anxiety	2.41 ± 0.54	2.53 ± 0.56	1.69	>0.05
Hostility	1.64 ± 0.45	1.62 ± 0.48	0.23	>0.05
Terror/Fear	1.41 ± 0.41	1.46 ± 0.40	0.74	>0.05
Paranoia	1.40 ± 0.49	1.39 ± 0.55	0.28	>0.05
Psychosis	1.36 ± 0.41	1.33 ± 0.59	0.83	>0.05

**Table 7:** Comparison of anxiety and depression in patients with COVID-19 before and after intervention.

Group		GAD-7	PHQ-9
Control (*n* = 55)	Before intervention	8.73 ± 4.24	8.16 ± 3.92
	After intervention	7.78 ± 3.27 *	7.31 ± 3.93 *
Intervention (*n* = 55)	Before intervention	8.69 ± 4.17	8.96 ± 4.15
	After intervention	5.65 ± 2.17 *^,#^	5.75 ± 2.47 *^,#^

Note: Compared with the group before intervention treatment, * *p* < 0.05; Compared with the control group after intervention treatment, ^#^ *p* < 0.05.

**Table 8:** Comparison of SCL-90 factors in patients with COVID-19 before and after intervention.

	Control Group (*n* = 55)		Intervention Group (*n* = 55)	
	Before Intervention	After Intervention	Before Intervention	After Intervention
Somatization	1.85 ± 0.50	1.73 ± 0.44	1.72 ± 0.56	1.39 ± 0.43 *^,#^
Obsessive Compulsive symptoms	1.66 ± 0.56	1.67 ± 0.58	1.69 ± 0.53	1.64 ± 0.51
Interpersonal Relationships	1.68 ± 0.59	1.67 ± 0.55	1.65 ± 0.51	1.64 ± 0.49
Depression	1.86 ± 0.43	1.74 ± 0.44	1.84 ± 0.56	1.58 ± 0.40 *^,#^
Anxiety	2.41 ± 0.54	2.26 ± 0.54 *	2.53 ± 0.56	1.54 ± 0.46 *^,#^
Hostility	1.64 ± 0.45	1.60 ± 0.49	1.62 ± 0.48	1.54 ± 0.45 *^,#^
Terror/Fear	1.41 ± 0.41	1.37 ± 0.42	1.46 ± 0.40	1.40 ± 0.41 *^,#^
Paranoia	1.40 ± 0.49	1.38 ± 0.57	1.39 ± 0.55	1.34 ± 0.51
Psychosis	1.36 ± 0.41	1.36 ± 0.48	1.33 ± 0.59	1.35 ± 0.51

Note: Compared with the group before intervention treatment, * *p* < 0.05; Compared with the control group after intervention treatment, ^#^ *p* < 0.05.

**Table 9:** Comparison of the length of hospital stay for COVID-19 patients.

	*n*	Length of Hospital Stay (Days)	*p*
Control group	55	9.67 ± 2.18	>0.05
Intervention group	55	9.01 ± 2.63	

## Data Availability

Data for this study are available from the corresponding author upon reasonable request.
